# How does increasingly plainer cigarette packaging influence adult smokers’ perceptions about brand image? An experimental study

**DOI:** 10.1136/tc.2008.026732

**Published:** 2008-09-30

**Authors:** M A Wakefield, D Germain, S J Durkin

**Affiliations:** Centre for Behavioural Research in Cancer, The Cancer Council Victoria, Victoria, Australia

## Abstract

**Background:**

Cigarette packaging is a key marketing strategy for promoting brand image. Plain packaging has been proposed to limit brand image, but tobacco companies would resist removal of branding design elements.

**Method:**

A 3 (brand types) × 4 (degree of plain packaging) between-subject experimental design was used, using an internet online method, to expose 813 adult Australian smokers to one randomly selected cigarette pack, after which respondents completed ratings of the pack.

**Results:**

Compared with current cigarette packs with full branding, cigarette packs that displayed progressively fewer branding design elements were perceived increasingly unfavourably in terms of smokers’ appraisals of the packs, the smokers who might smoke such packs, and the inferred experience of smoking a cigarette from these packs. For example, cardboard brown packs with the number of enclosed cigarettes displayed on the front of the pack and featuring only the brand name in small standard font at the bottom of the pack face were rated as significantly less attractive and popular than original branded packs. Smokers of these plain packs were rated as significantly less trendy/stylish, less sociable/outgoing and less mature than smokers of the original pack. Compared with original packs, smokers inferred that cigarettes from these plain packs would be less rich in tobacco, less satisfying and of lower quality tobacco.

**Conclusion:**

Plain packaging policies that remove most brand design elements are likely to be most successful in removing cigarette brand image associations.

In the face of comprehensive restrictions on tobacco advertising and promotion, tobacco packaging has become the primary vehicle for communicating brand image.^1^ Through the use of colour, fonts, images and trademarks, cigarette packs project a brand image that says something about the user of the product. Commonly referred to as a “badge product”, the user often associates with the identity and personality of the brand image.^2^ ^3^ Unlike most other consumer products, cigarette packs remain with users once opened and are repeatedly displayed in social situations, thereby serving as a direct form of mobile advertising for the brand.

In countries such as Australia where traditional forms of advertising are banned, packaging now serves as the main vehicle for tobacco marketing. Accordingly, Australian tobacco companies have experimented with producing more colourful and varied packs, as well as designs to pique curiosity. For example, British American Tobacco (BAT) Australia experimented with its trademark design on packs of Benson and Hedges and Winfield cigarettes in 2002–3^4^ and introduced split Dunhill packs (so-called “kiddie packs”) in 2006,^5^ ^6^ by which two low-consumption smokers could more easily procure and split apart a single pack for their own use. Some brands have also begun to incorporate the colour schemes of graphic health warnings into the overall colour and design of the entire pack, causing the warnings to become less salient since they blend in with the overall pack design (Kylie Lindorff, Quit Victoria, personal communication, July 2008). Bans on traditional forms of tobacco advertising and promotion also lead to a more critical role for cigarette packaging at the point of sale, where packs are designed to allow brand families to better stand out at the cash register.^2^ ^7^ These point-of-sale tobacco advertising and cigarette displays create an enticing in-store presence for youth,^8^^–^10 and a cue to prompt adult smokers to purchase.^11^

In response to these developments, proposals to introduce “plain” cigarette packaging have emerged whereby packs would be stripped of colours, brand imagery, corporate logos and trademarks and manufacturers would be permitted to print only the brand name in a mandated size, font and location, in addition to required health warnings and other legally mandated information such as toxic constituents, tax seals or pack contents.^12^ ^13^ Aside from denying that the pack is a form of advertising, a key argument of the tobacco industry against plain packaging is that it would amount to trademark infringement and unjustifiably encumber the use of trademarks in the course of trade, violating several international trade and intellectual property agreements such as the Trade-Related Aspects of International Property Rights (TRIPS) Agreement 1994, the North American Free Trade Agreement 1994 (NAFTA) and the Paris Convention for the Protection of Industrial Property 1883.^13^^–^15 However, as Freeman and colleagues^13^ argue, the industry’s interpretation of these agreements is selective, as each of these treaties contains specific exemptions allowing necessary measures to be adopted to protect public health and to protect the public interest.

Research by the tobacco industry has shown that the design of a cigarette pack can not only generate powerful images about the type of person who might typically smoke the brand, but also provide cues about the sensory perceptions of the smoke which may be expected from a particular cigarette. For example, given identical cigarettes to try, men and women rated the sensory experience of smoking a cigarette differently depending on the brand name given to the cigarette, with women rating the attributes of the smoke more positively when assigned a feminine brand name and men rating it more positively if it had a masculine brand name.^16^ Similarly, sensory perceptions of cigarettes can be manipulated simply by changing the colour or shade of colour on a pack, through a process called “sensation transfer”. Package testing for Camel Filter cigarettes revealed that increasing the amount of white space on the pack and lightening brown colour tones reduced the perception of the cigarette’s strength when the cigarette was smoked.^17^ Research conducted by Philip Morris USA also indicated strong sensation transfer effects when testing identical Marlboro Ultra Light cigarettes placed in either a blue or red pack. Although the cigarettes were exactly the same, those placed in the red packs were perceived to be “harsher” than those in the blue packs, while cigarettes in the blue packs were rated as “too mild”, “not easy drawing” and “burned too fast”.^18^

Previous experimental studies examining the potential impact of plain packaging have shown that health warnings are more noticeable when presented on a plain cigarette pack,^19^^–^22 and that plain packs detract noticeably from brand imagery established by cigarette brands.^20^ ^21^ ^23^ To our knowledge, no research has examined the effects of plain packaging on smoker’s perceptions of taste, strength or quality of the product, and little attention has so far been focused on the testing of different plain pack versions against each other, examining the impact of branded fonts and other brand elements on packs.

This study aims to provide research evidence to assist the selection of plain pack designs that would promote the least positive attributes about smoking for smokers. We hypothesise that smokers will rate an original branded pack more positively than their plain pack counterparts, and that plain packs with progressively fewer brand-associated elements will be rated more negatively.

## METHODS

### Design

This study employed a 3 (brand types) × 4 (degree of plain packaging) between-subject experimental design using an internet online method to expose adult smokers to one randomly selected cigarette pack, after which respondents completed ratings of the pack.

### Sample

A market research company was commissioned to undertake the administration of the survey. A sampling frame of adults aged 18–49 years was sourced from an existing national online panel. The panel members were originally sourced from various methods including computer-assisted telephone interviews and face-to-face market research, during which participants supplied their email address and gave permission to be contacted by email to participate in future research as well as through online marketing and other online databases. The panel was broadly representative of Australian Bureau of Statistics norms in relation to geographical location, income and age. Using Cohen’s power calculations,^24^ we estimated that a sample size of 780 would allow the detection of small-to-medium effect sizes for main effects (<0.50; p = 0.05; power = 0.99).

### Procedure

Eligible participants in the panel were sent an email that included a web link to the survey, inviting them to participate in a study about their opinions of a brand with which they might be familiar. Respondents were given a chance to win one of 10 AU$100 shopping vouchers as an incentive to participate. A reminder email was sent 5 days after the initial email, and a final reminder was sent a further 5 days later. Upon accessing the survey website, demographic information was collected including sex, age, level of educational attainment, postcode and whether they were daily or weekly smokers of manufactured cigarettes. Respondents who said they smoked less than weekly or not at all and/or those outside the age criteria were excluded from further participation in the study.

Eligible respondents were then randomly allocated to view one of 12 pack conditions that varied by brand and extent of plain packaging. The three brands were the three most popular Australian brand variants among adult smokers (Winfield Blue 25s; Peter Jackson Rich 30s; Longbeach Rich 40s).^25^ Previous tobacco company research on packaging perceptions has found that particular pack colours are associated with specific perceptions—for example, red connotes strength in taste, blue suggests a lighter strength cigarette and white connotes the freshest and lightest cigarettes of all.^2^ ^3^ As much is already known about the effects of specific pack colours, the current study did not test different pack colours but presented all plain packs in a cardboard brown colour previously demonstrated to elicit negative responses.^26^ ^27^ The four pack design conditions were:

- Original pack: an existing pack one could purchase today.
- Plain pack 1: a generic cardboard brown pack that maintains a branded font (ie, original font size, style and position) and positioning of brand/descriptor.
- Plain pack 2: a generic cardboard brown pack with the brand name in a standard font in a prominent position on the pack with descriptor information in a standard font at the bottom.
- Plain pack 3: a generic cardboard brown pack with the brand name in a smaller standard font positioned at the bottom and “(xx number) cigarettes” in a larger font in a prominent position on the pack.

**Figure 1:**
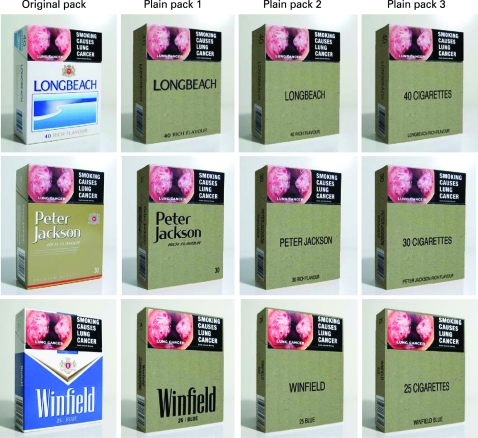
Original and plain packs for each brand.

All pack conditions had the same graphic health warning visible on the top of the face of the pack as required by Australian Government legislation.^28^ In light of the tobacco industry’s argument that enforcement of plain packaging would amount to trademark infringement and unjustifiably encumber the use of trademarks in the course of trade, during the development of our hypotheses and the designs of generic packs for testing, legal advice from an intellectual property lawyer was sought to ensure that we would be testing packs that could realistically be introduced into the market place without impeding trademark laws. Figure 1 displays each of the 12 pack conditions.

After viewing their assigned pack, respondents completed ratings of the pack in relation to perceived attributes of the brand, perceived attributes of smokers of the brand and expected taste/quality of the cigarette. The assigned pack was present on the screen as the smoker completed each of the ratings.

### Questionnaire

Attributes to be rated were modified from past tobacco industry packaging studies where smokers were asked to rate cigarette packs on attractiveness, brand imagery characteristics and perceived sensory attributes.^29^ ^30^ In the current study, respondents were asked to rate the cigarette pack they were shown in relation to: brand image (the mental associations that are stimulated by the pack’s appearance alone); smoker attributions (anticipated personality/character type of the typical person who might be expected to regularly smoke the pack displayed); and inferred smoking experience (the type of smoking experience which might be anticipated from a cigarette contained in the displayed pack).

When viewing the cigarette pack, respondents were asked to rate the following phrases describing attributes of the cigarette pack shown from 0 (not at all well) to 10 (extremely well). “This pack …”: “is a popular brand among smokers”; “has an attractive looking pack”; “is good value for money”; “is an exclusive/expensive brand”; and “is a brand you might try/smoke”. Looking at the same pack, respondents were then asked to rate a number of attributes of typical smokers of the pictured cigarette pack from 0 (not at all) to 10 (extremely well). “A typical smoker of this pack is …”: “trendy/stylish”; “young”; “masculine”; “lower class”; “sociable/outgoing”; “older/mature”; and “confident/successful”. Finally, looking at the same pack, respondents were asked to think about how a cigarette from the pictured pack might taste, and to rate the following descriptions on how well they relate to the pack shown from 0 (not at all) to 10 (extremely). “These cigarettes would taste …”: “rich in tobacco flavour”; “low in tar and nicotine”; “of cheap tobacco”; “satisfying”; “like a light cigarette”; “of the highest quality tobacco”; and “harsh on the throat”. Within each of the questions, attributes were presented randomly to avoid order effects.

Once the final question was completed, respondents submitted their responses to the survey, were thanked for their participation and told they had been entered in the draw for the shopping vouchers.

### Statistical analysis

Analysis of variance and χ^2^ tests were used to check that random assignment yielded equivalent groups with respect to smoking history and demographic characteristics. Preliminary analyses indicated that survey responses on the 11-point response scale were not normally distributed. Responses were skewed at two points on the scale: at 0 (indicating disagreement) and at 5 (indicating moderate agreement). We therefore dichotomised responses to permit statistical analysis, with responses from 0 to 4 categorised together to reflecting “disagreement to low agreement” and responses from 5 to 10 reflecting “moderate to high agreement”. Differences between pack conditions were assessed using logistic regression analysis to generate odds ratios and confidence intervals.

## RESULTS

### Sample characteristics and group assignment

Overall, 813 regular smokers resident in Australia completed the study procedure, yielding a response rate of 22% of all those sent email invitations. In total, 62% of smokers were female, 81% were aged 30 years or older, 36% had completed Year 11 secondary education or less, 45% had completed Year 12 education or some tertiary, and 19% had completed a tertiary qualification. Just under half (47%) smoked >15 cigarettes per day on average. Respondents were also classified by postcode of residence into four levels of social advantage/disadvantage based on the Socio-Economic Index for Areas (SEIFA) developed by the Australian Bureau of Statistics.^31^ Just under one-quarter (21%) of respondents lived in areas of low advantage, while 27% were living in areas of high advantage. Overall, 17% of participants were assigned to view a brand that they smoked. Table 1 shows that demographic and smoking characteristics of the respondents did not vary significantly across the different pack conditions. An average of 203 respondents (minimum 176; maximum 219) were randomly allocated to each of the four pack conditions.

**Table 1:** Demographic and smoking characteristics of participants by pack condition

	Original(n = 176)	Plain pack 1(n = 219)	Plain pack 2(n = 199)	Plain pack 3(n = 219)	p Value
Male (%)	38.6	38.4	35.2	40.2	0.765
Age (%)					0.206
18–29 years	18.2	17.4	24.6	17.8	
30+ years	81.8	82.6	75.4	82.2	
Education (%)					0.684
Year 11 or less	31.7	36.8	35.6	39.0	
Year 12/some tertiary	50.6	42.0	45.2	43.0	
Tertiary	17.7	21.2	19.1	18.0	
Socioeconomic status (%)					0.409
SEIFA 1 (lowest advantage)	23.3	19.6	20.6	20.8	
SEIFA 2	18.2	21.0	18.1	20.4	
SEIFA 3	30.7	36.5	37.2	27.3	
SEIFA 4 (highest advantage)	27.8	22.8	24.1	31.5	
Consumption (%)					0.355
1–10 cigs/day	29.0	27.9	24.6	27.9	
11–15 cigs/day	24.4	29.7	22.1	26.9	
16–20 cigs/day	25.0	16.9	21.1	22.8	
21–25 cigs/day	11.4	13.7	14.6	10.5	
26 + cigs/day	10.2	11.9	17.6	11.9	
Brand seen is brand smoked	19.3	17.4	15.6	15.5	0.727

SEIFA, Socio-Economic Index for Areas.

### Effect of pack condition on perceptions

The results of fitting a logistic regression model with an interaction between pack condition and brand to predict pack perceptions indicated that there were no interactions between these two variables. Therefore, in the following analyses, the results for the three brands were aggregated. Table 2 shows that for all brands combined, Plain pack 1, which preserved the placement and font of brand names and brand variants, was perceived as less attractive than the original branded pack, and smokers of the pack were perceived as less sociable and outgoing than smokers of the original pack. There was also a trend for smokers of Plain pack 1 to be perceived as less trendy and stylish than smokers of the original pack. On all other dimensions, Plain pack 1 was rated as similar to the original branded pack.

**Table 2:** Bivariate logistic regression analyses comparing percentage of smokers who agreed with rated attributes, by pack condition†

	Original		Plain pack 1		Plain pack 2		Plain pack 3		OR for linear trend
	%	OR	%	OR	%	OR	%	OR	
*Brand/pack characteristics*									
Popular brand among smokers	83.5	1	78.1	0.70	75.9	0.62‡	67.1	0.40***	0.75***
Attractive looking pack	50.0	1	34.7	0.53**	31.2	0.45***	32.0	0.47***	0.79***
Value for money	56.8	1	55.7	0.96	50.8	0.78	49.3	0.74	0.90‡
Exclusive/expensive brand	39.8	1	44.7	1.23	38.2	0.94	40.2	1.02	0.97
Brand you might try/smoke	59.1	1	55.7	0.87	53.3	0.79	51.6	0.74	0.91
									
*Smoker characteristics*									
Trendy/stylish	47.2	1	38.4	0.70‡	34.2	0.58*	32.0	0.53**	0.81**
Young	55.1	1	52.1	0.88	41.2	0.57**	47.9	0.75	0.88*
Masculine	58.0	1	59.8	1.08	55.8	0.92	42.9	0.55**	0.81***
Lower class	52.8	1	54.3	1.06	50.3	0.90	53.0	1.01	0.99
Sociable/outgoing	68.8	1	55.7	0.57**	51.8	0.49***	49.3	0.44***	0.78***
Older/mature	67.0	1	65.8	0.94	61.8	0.80	55.7	0.62*	0.85*
Confident/successful	51.7	1	51.6	1.00	42.7	0.70	43.4	0.72	0.87*
								
*Perceived sensory perceptions*									
Rich in tobacco	76.1	1	70.8	0.76	64.8	0.58*	67.1	0.64*	0.86*
Low in tar and nicotine	44.9	1	38.4	0.76	33.7	0.62*	33.3	0.61*	0.85*
Tastes of cheap tobacco	54.5	1	47.0	0.74	50.3	0.84	50.7	0.86	0.97
Satisfying	72.7	1	65.3	0.71	64.8	0.69	61.2	0.59*	0.86*
Like a light cigarette	47.2	1	41.1	0.78	43.2	0.85	39.7	0.74	0.92
Of the highest quality tobacco	60.8	1	59.8	0.96	51.8	0.69‡	50.7	0.66*	0.85*
Harsh on throat	50.6	1	48.9	0.93	54.3	1.16	52.5	1.08	1.05

*p<0.05; **p<0.01; ***p<0.001.

†Scored 5 or more on a scale from 0 (not at all) to 10 (extremely).

‡p<0.10.

Compared with the original branded pack, Plain pack 2, which standardised the placement and font of the brand name and relinquished the brand variant to standard type at the bottom of the pack, was rated as less attractive, and smokers of the brand were rated as less trendy and stylish, less young and less sociable and outgoing. In addition, compared with those who viewed the original pack, fewer smokers who viewed Plain pack 2 thought the cigarettes would be low in tar, fewer thought the cigarettes would be rich in tobacco and of the highest quality tobacco. There was also a tendency for Plain pack 2 to be rated as less popular than the original pack.

Compared with the original branded pack, Plain pack 3, where the brand name and variant appeared only in small standard type at the bottom of the pack, was perceived as being less popular and less attractive, and smokers of the brand were perceived to be less trendy and stylish, less masculine, less sociable or outgoing and less mature. Compared with those who viewed the original pack, significantly fewer smokers who viewed Plain pack 3 thought the cigarettes would be low in tar, rich in tobacco, satisfying to smoke and of the highest quality tobacco.

**Figure 2:**
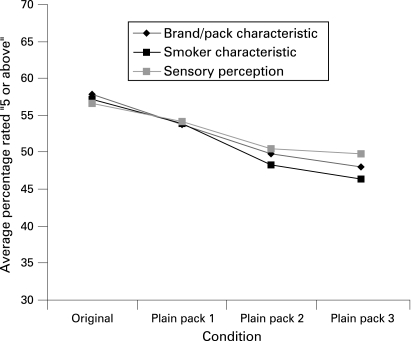
Smokers’ ratings by pack condition.

Table 2 also shows that, for most of these mentioned attributes, there was a significant linear decline in the degree of favourable ratings as pack branding design information reduced. To graphically represent this (fig 2), we combined the variables within each of the three categories of ratings (ie, brand/pack characteristics; smoker characteristics; perceived sensory perceptions) after testing the strength of correlations within each category (brand/pack characteristics: Cronbach’s α = 0.72; smoker characteristics: Cronbach’s α = 0.87; perceived sensory perceptions: Cronbach’s α = 0.74).

## DISCUSSION

This study suggests that cigarette packs that display progressively fewer branding design elements and presented in a generic brown colour are perceived increasingly unfavourably by smokers. Even though all plain packs substituted a cardboard brown colour for the original pack colour, the removal of additional design elements produced measurable decrements in smokers’ appraisals of the packs, the smokers who might smoke such packs, and the inferred experience of smoking a cigarette from these packs. Although we did not explicitly test this, it is possible that the gradual removal of design elements may also have served to increase the salience of the pictorial health warnings as suggested in earlier research,^19^^–^22 and this would be a desirable additional outcome.

**What this paper adds**

- Plain tobacco packaging has been proposed as a means to limit brand imagery, but little research has been undertaken to guide decision-making about which packaging brand design elements drive brand appeal for smokers.
- This experimental study found that plain packs with increasingly fewer brand design elements are perceived increasingly unfavourably in terms of smokers’ appraisals of the packs, the smokers who might smoke such packs, and the inferred experience of smoking a cigarette from these packs.
- This implies that tobacco control policies should aim to remove as many brand design elements as possible.

There are a number of study limitations that should be mentioned. First, the use of an 11-point response scale produced an irregular response distribution and we needed to dichotomise responses to conduct analysis. In future studies a more usual 5-point Likert scale with named response options would be preferred. However, even though we dichotomised responses, we were still able to detect differences between pack conditions. Second, although we tested three variations of plain packs, each condition removed several design elements at one time and we were not able to determine which specific brand elements most contributed to deteriorations in smoker perceptions of the packs. Other study designs such as fractional factorial design where a single brand element can be manipulated may be better suited for this more finely-tuned purpose.^32^ However, our study has shown that, in aggregate, smokers perceive plain cardboard brown packs with fewer branding elements less favourably, and this applied to the three brand variants most commonly smoked in Australia. Along the same lines, we may have obtained different results using packs with different background colours other than the cardboard brown we selected. However, the colour selected was chosen purposively as a result of previous research where it elicited negative perceptions.^26^ Third, our study displayed packs via an internet image which did not permit smokers to handle the pack. This reduction in pack-related information might have been expected, however, to understate the brand design elements, leading to underestimates of differences between pack conditions. Thus, our study results may be conservative. In addition, confidence in the validity of responses would have been stronger if a rationale was provided to respondents for the existence of the plain packs. Finally, the internet method of survey administration may have allowed some smokers to seek the input of others into the responses they gave. However, if this occurred, the randomised design would have meant that this kind of interference in responses was equally distributed across conditions. As our sample was sourced from an existing online panel with a consequent low response rate, respondents were not representative of the general population in terms of demographic characteristics. However, this was an experimental study rather than a population survey, and the online method was simply used to recruit smokers to the experiment and randomise them to one of the experimental conditions. Randomisation was successful as judged by the fact that groups did not differ in composition. Overall, our internet method of stimulus presentation provided a simple inexpensive experimental method for obtaining responses from a large sample size to randomly-presented stimulus packs.

With a likely acceleration in the rate of comprehensive restrictions on tobacco advertising and promotion as countries strive to meet their responsibilities under the Framework Convention on Tobacco Control (FCTC),^33^ tobacco packaging will assume even greater importance internationally as a promotional vehicle for driving brand image.^3^ Plain packaging measures remain an important yet relatively under-explored component of tobacco control legislation designed to comprehensively eliminate all forms of tobacco advertising and promotion. In their review, Freeman and colleagues^13^ conclude that trademark laws and international trade laws do not preclude mandating the removal of brand design elements on tobacco packs and that plain packaging could and should be pursued under the FCTC. Our research extends the existing evidence base by demonstrating not only that plain packs are perceived unfavourably by smokers, but that plain packs with the least brand design elements have the least appeal. Further research to quantify more carefully the effects of specific design elements on brand perceptions—including among youth at risk for smoking—would provide helpful guidance for future policy development.
